# Sleep Quality and Sleepiness Among Veterinary Medical Students Over an Academic Year

**DOI:** 10.3389/fvets.2019.00119

**Published:** 2019-04-17

**Authors:** Michael T. Nappier, Lara Bartl-Wilson, Tiffany Shoop, Shelby Borowski

**Affiliations:** ^1^Department of Small Animal Clinical Sciences, Virginia-Maryland College of Veterinary Medicine, Virginia Tech, Blacksburg, VA, United States; ^2^Center for Excellence in Teaching and Learning, Virginia Tech, Blacksburg, VA, United States; ^3^Department of Population Health Sciences, Virginia-Maryland College of Veterinary Medicine, Virginia Tech, Blacksburg, VA, United States

**Keywords:** sleep quality, sleepiness, veterinary medical students, Pittsburgh sleep quality index, epworth sleepiness scale

## Abstract

Good sleep health is a key component to good personal well-being. It has been postulated that veterinary students have poor sleep health, but few measurements have been undertaken. This study measured Pittsburgh Sleep Quality Index and Epworth Sleepiness Scale values at multiple points throughout an academic year for students in a veterinary medical curriculum. Students were found to have overall poor sleep quality and above average to excessive daytime sleepiness. Further investigation is necessary to determine specific causes as well as what action can be taken to improve student sleep health.

## Introduction

Good sleep health is a critical and integral component of good personal health and wellness (1, 2). The National Sleep Foundation recommends 7 to 9 h of sleep for young adults (18–25 years old) and seven to 8 h of sleep for adults (26–64 years old) (3). However, many Americans self-report sleeping less than the recommended amount of sleep (4). Sleeping less than the recommended number of hours has been associated with a range of health problems including hypertension, diabetes, obesity, cardiovascular disease, depression, and suicide (5, 6).

Personal wellness and health are serious and ongoing issues in veterinary medicine (7). Veterinarians are an at-risk population for poor personal wellness which can result in burnout, substance abuse, depression, anxiety, and suicide (8, 9). In one study, 88% of practicing veterinarians who responded stated that veterinary medicine is very stressful, 66% stated that they had clinical levels of depression, and 20% reported seriously considering suicide (10). Veterinary students are not exempt from these problems. A survey of veterinary students revealed a bevy of wellness problems: headaches, sleep disturbance, overly busy thinking, inability to concentrate, increase or decrease in food intake, procrastination, depression, feeling overwhelmed, and chronic tiredness (11).

It has been generally well documented that college students often have poor sleep health and frequently do not get the recommended amount of sleep for their age (12, 13). Students who have poor sleep health have been found to have poor health and wellbeing (14). Poor sleep health has also found to result in decreased academic performance, poor procedural and declarative learning, and reduced learning capacity (15).

Poor sleep health has been well documented in human medical students. Medical students in Saudi Arabia were documented to sleep only an average of about 6 h a day (16). In Brazil, medical students were found to have significantly poorer sleep quality than counterparts in other academic programs (17). This was particularly true during the first 2 years of their program (17). Medical students with poor sleep health have been shown to exhibit higher levels of burnout and poorer personal well-being (18). Sleep deprivation in senior medical students and first year medical residents has shown a decrease in motor coordination, memory, and informational processing capabilities (19, 20).

Veterinary students, therefore, fall into a doubly at-risk category of both being students in a medical education program and being in the field of veterinary medicine. However, almost no information is available about the sleep health of veterinary students. The lone available study in this area revealed that >50% of students reported getting less than the recommended amount of sleep as well as having significant daytime sleepiness (21). In that study 28% of students reported having trouble sleeping, 42% rated their sleep quality as fair or poor, and 68% reported driving while drowsy (21).

## Materials and Methods

### Procedure and Sample

Veterinary students enrolled in a large Southeastern university participated in the study. Participants were recruited though a listserv. Data were collected via a web-based questionnaire. Veterinary students were emailed the questionnaire at five differing time points over the course of the year: September, November, December, February, and May. Time points were chosen to roughly coincide with beginning, middle, and end of both fall and spring semesters. Participants were not compensated for their participation. This study was carried out in accordance with the recommendations of the Virginia Tech Institutional Review Board, Protocol #17-712, with written informed consent from all subjects. All subjects gave written informed consent in accordance with the Declaration of Helsinki. The protocol was approved by the Virginia Tech Institutional Review Board.

The sample consisted of 312 students currently enrolled in veterinary school. The majority of participants were female (*n* = 232, 74.4%). Eighty-two (26.3%) were enrolled in their first year of veterinary school, 78 (25%) were in their second year, 84 (26.9%) were in their third year, and 68 (21.8%) were in their fourth year.

The final sample size for the sleep quality model was 307 veterinary students with a total of 781 observations. The number of time points completed from students ranged from 1 to 5, with an average of 2.6 time points (SD = 1.5, Mdn = 2) completed per person. The final sample size for the sleepiness model was 308 veterinary students with a total of 790 observations.

### Measures

#### Year in Veterinary School

Participants reported their year in veterinary school by responding to the question “What year are you in veterinary school?”

#### Sleep Quality

Participants' sleep quality was assessed using the 19-item self-report Pittsburgh Sleep Quality Index, which measures retrospective sleep quality over the past 30 days (22). This index is comprised of seven components of sleep quality: Subjective sleep quality (1 item), sleep latency (2 items), sleep duration (1 item), habitual sleep efficiency (3 items), sleep disturbances (9 items), use of sleeping medication (1 item), and daytime dysfunction (2 items). Each component was scored 0 to 3 and summed to provide a global score for sleep quality; thus, possible global scores range from 0 to 21. Higher global scores indicated poorer sleep quality, more specifically, a score of >5 indicated poor sleep quality. Data for any component were not allowed to be missing. Therefore, participants with any missing component score were omitted from the sleep quality analyses. Scores obtained from the PSQI had a Cronbach's alpha reliability coefficient of 0.74.

#### Sleepiness

Participants' daytime sleepiness was assessed using the 8-item Epworth Sleepiness Scale (23). Participants rated the chances they would doze off or fall asleep in eight different situations using a 4-point Likert scale, ranging from 0 (would never doze) to 3 (high chance of dozing). The eight items were summed to create a total sleepiness scores; thus, possible scores range from 0 to 24. Higher sleepiness scores indicated more daytime sleepiness. “Lower normal daytime sleepiness” scores range from 0 to 5, “high normal daytime sleepiness” scores range from 6 to 10, while “excessive daytime sleepiness” scores range from 11 to 24. Data for any item were not allowed to be missing. Therefore, participants with any item missing were omitted from the sleepiness analyses. Scores obtained from the ESS had a Cronbach's alpha reliability coefficient of 0.81.

### Data Analysis

For the nested data (repeated measures within individuals), multilevel models were analyzed in SPSS MIXED using restricted maximum likelihood (24). Separate models were conducted for sleep quality and sleepiness, so sample sizes in both models vary slightly due to missing data. Level-1 predictors in the models are time-varying. The time point in the academic year was the time-varying predictor. Time 1 (i.e., the beginning of the academic year in September) was entered in both models as a predictor where T1 = 0, to represent the intercept of the outcome. Level-2 variables in the models are time-invariant. In the analyses, year in veterinary school was the level-2 time-invariant predictor.

Data were examined for any violations of normality and no violations were present. Sleep quality and sleepiness scores across the five time points were plotted separately for each veterinary student. Examination of these individual scatterplots indicated that sleep quality and sleepiness across the academic year was linear. Based on model fit statistics and the number of time points, random-intercept models were estimated for sleep quality and sleepiness.

In the multilevel models, the fixed effects are the results for the typical first, second, third, and fourth year vet student. First year students were used as the reference group for both models. The *intercept* estimate represents the mean score for the typical first year student. The estimates for second, third, and fourth year students represent the difference in intercept scores from the typical first year student. The *time (1*^*st*^ *year)* estimate represents the slope for the typical first year student, and all other *time x year* estimates represent the difference in slopes from typical first year student. The random effects describe the variability at level-1 and level-2.

## Results

### Preliminary Analyses

Table 1 displays the means and standard deviations across the five time points for sleep quality and sleepiness.

**Table 1:** Descriptive statistics for sleep quality and sleepiness by time point for all veterinary students.

	**Sleep Quality**		**Sleepiness**	
**Time Point**	***M***	***SD***	***M***	***SD***
1	7.05	3.33	9.24	4.41
2	7.43	3.66	9.79	4.58
3	7.38	3.23	9.68	4.77
4	7.37	3.35	9.41	4.50
5	6.78	3.53	8.63	4.54

For sleep quality, total scores ranged from 1 to 21, with a mean >5 at every time point. Of the 781 valid sleep quality observations, 64.1% (*n* = 501) had a score of >5. There were no significant gender differences in sleep quality (*p* = 0.469). The average sleep quality score for males was (*M* = 7.26, *SD* = 3.12) and females was (*M* = 7.21, *SD* = 3.48).

For sleepiness, total scores ranged from 0 to 22, with a mean between 5 and 10 at every time point. Of the 790 valid sleepiness observations, 41.3% (*n* = 326) had a score between 11 and 24. There were significant gender differences in sleepiness (*p* = 0.006). Females (*M* = 9.65, *SD* = 4.55) reported significantly higher sleepiness scores compared to males (*M* = 8.57, *SD* = 4.49).

Correlations between the seven sleep quality components, global sleep quality score and sleepiness score can be found in Table 2. Means and standard deviations are also reported (Table 2).

**Table 2:** Means, standard deviations, and correlations.

**Variable**	***M***	***SD***	**1**	**2**	**3**	**4**	**5**	**6**	**7**	**8**	**9**
1. Subjective sleep quality	1.27	0.69	–								
2. Sleep latency	1.39	0.99	0.47^***^	–							
3. Sleep duration	0.97	0.74	0.48^***^	0.23^***^	–						
4. Habitual sleep efficiency	0.31	0.66	0.36^***^	0.29^***^	0.48^***^	–					
5. Sleep disturbances	1.26	0.50	0.38^***^	0.35^***^	0.18^***^	0.27^***^	–				
6. Use of sleeping medication	0.46	0.89	0.27^***^	0.30^***^	0.17^***^	0.21^***^	0.25^***^	–			
7. Daytime dysfunction	1.55	0.84	0.44^***^	0.27^***^	0.34^***^	0.21^***^	0.35^***^	0.20^***^	–		
8. Global sleep quality	7.22	3.40	0.75^***^	0.69^***^	0.64^***^	0.60^***^	0.57^***^	0.57^***^	0.63^***^	–	
9. Sleepiness	9.41	4.56	0.33^***^	0.18^***^	0.33^***^	0.20^***^	0.25^***^	0.16^***^	0.49^***^	0.44^***^	–

*Means and standard deviations presented are from all observations across time points*.

*** *p < 0.001*.

### Sleep Quality (RQ1)

**Figure 1:**
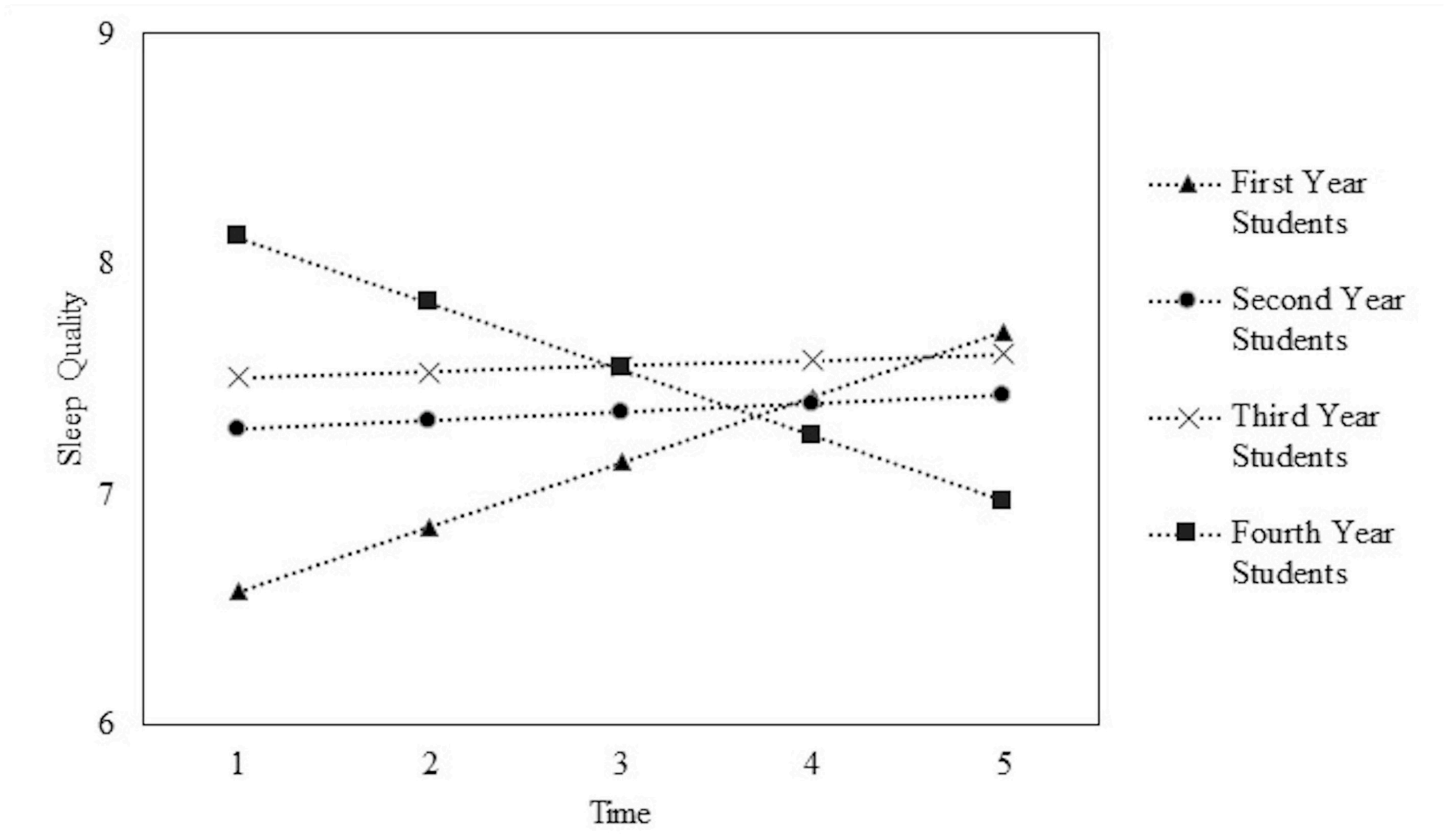
Plot of average time courses for first, second, third, and fourth year veterinary students sleep quality scores across the academic year. Time 1 = beginning of the academic year and Time 5 = end of the academic year.

The typical trajectory of sleep quality for veterinary students over the academic year are displayed in Figure 1 and estimates are displayed in Table 3. At the beginning of the academic year, fourth year students (dashed line with square markers) have the worst sleep quality score (8.12 units) compared to other students. Third year students (dashed lines with x markers) have an initial sleep quality score of 7.51 units, followed by second year students (dashed line with circle markers) with an initial value of 7.28 units. First year students (dashed line with triangle markers) had the best sleep quality score (6.58 units) compared to other students at the beginning of the academic year (Figure 1).

**Table 3:** Parameters estimates for models predicting sleep quality and sleepiness across the academic year.

	**Sleep Quality**			**Sleepiness**		
	**Est**	***SE***	***t***	**Est**	***SE***	***t***
**Fixed Effects**						
Intercept (1st year students)	6.578	0.374	17.57^***^	8.747	0.496	17.63^***^
2nd year students	0.705	0.527	1.34	0.947	0.710	1.33
3rd year students	0.930	0.507	1.83	0.506	0.677	0.75
4th year students	1.540	0.564	2.73^**^	1.431	0.749	1.91
Time (1st year)	0.282	0.103	2.75^**^	0.267	0.155	1.72
Time x 2nd year	−0.244	0.141	−1.73	−0.312	0.216	−1.45
Time x 3rd year	−0.256	0.139	−1.85	−0.192	0.210	−0.91
Time x 4th year	−0.569	0.158	−3.61^***^	−0.486	0.241	−2.02^*^
**Random EFFECTS**						
Between-Person variance (level-2)	8.865	0.862	10.28^***^	13.304	1.459	9.12^***^
Within-Person variance (level-1)	2.821	0.231	12.18^***^	6.686	0.648	10.32^***^

*Time is coded as 0 = Time 1. First year students are the reference group*.

* p < 0.05;

** p < 0.01;

*** p < 0.001.

Over the academic year, first, second, and third year students showed an increase in their sleep quality scores. Fourth year students showed a decrease in their sleep quality scores. More specifically, for first year students, their sleep quality score increased an average of 0.28 units for each time point measured (i.e., every 1–2 months). Fourth year students, for every time point measured, had sleep quality scores decrease an average 0.29 units. Second and third year veterinary students increased an average of 0.04 and 0.03 units, respectively, in their sleep quality for every time point measured (Table 3).

### Sleepiness (RQ2)

**Figure 2:**
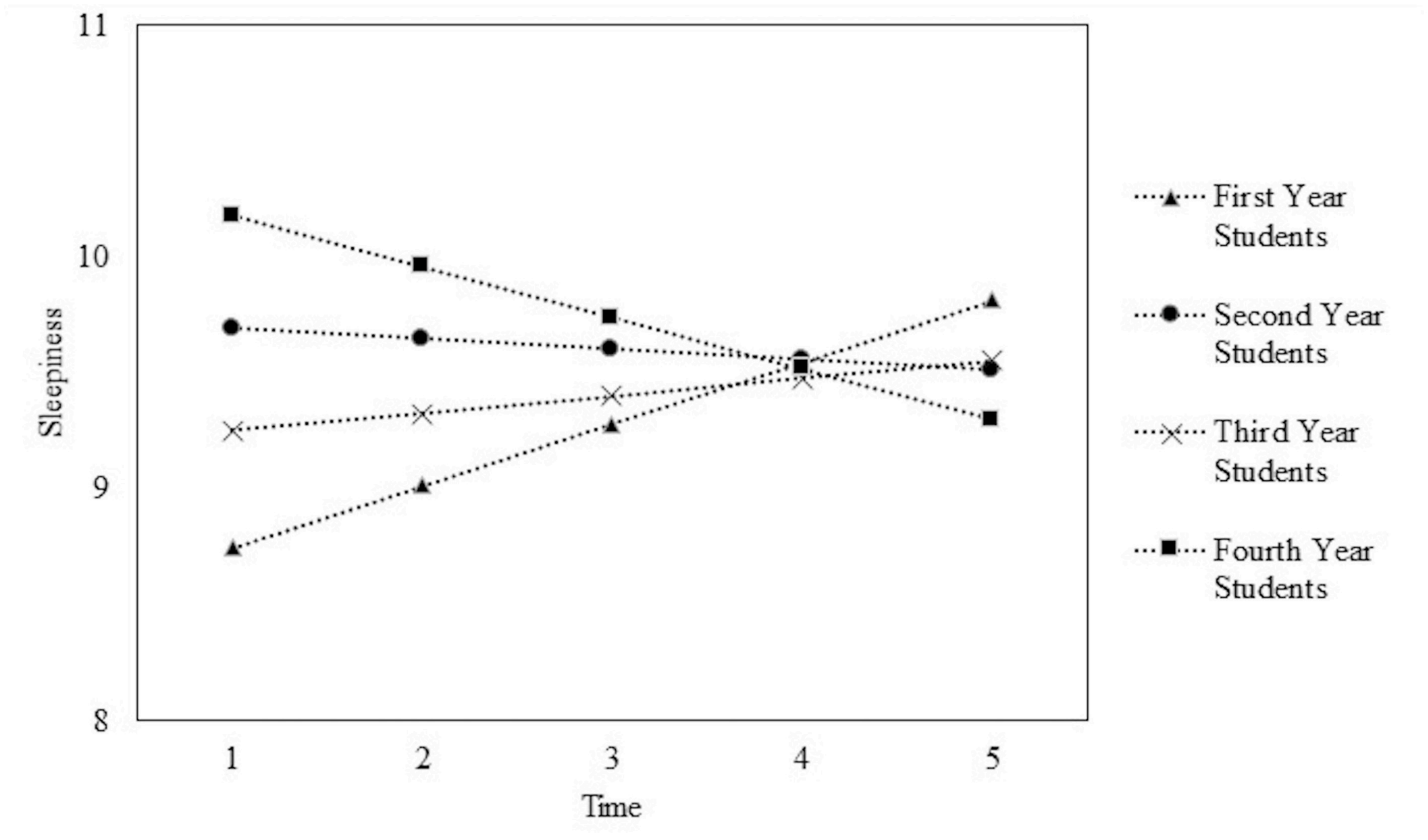
Plot of average time courses for first, second, third, and fourth year veterinary students sleepiness scores across the academic year. Time 1 = beginning of the academic year and Time 5 = end of the academic year.

The typical trajectory of daytime sleepiness for veterinary students over the academic year are displayed in Figure 2 and estimates are displayed in Table 3. At the beginning of the academic year, fourth year students have the highest sleepiness score (10.18 units), followed by second year students (9.69 units), and third year students (9.25 units). First year students had the lowest sleepiness score (8.75 units) (Figure 2).

Over the course of the academic year, first and third year students showed an increase in their sleepiness scores. For first year students, their sleepiness scores increased an average of 0.27 units for each time point measured. Third year students had a smaller increase with an average increase of 0.07 units. Second and fourth year students showed a decrease in their sleepiness score over the academic year. Fourth year students had an average decrease of 0.22 units for each time point measured. Second year students had a smaller decrease of 0.05 units.

## Discussion

As a whole, veterinary students in this study were found to have overall poor sleep quality and higher than normal daytime sleepiness throughout the academic year. This confirms overall suspicions of poor sleep quality and daytime sleepiness as a factor that is in play for overall student wellness in veterinary medical education. While these results are not surprising as it has already been reported that college students often have poor sleep health, it provides a baseline for overall sleep quality and daytime sleepiness in veterinary students that can be used for comparison. It also further reinforces and expands on the previously reported information available on sleep health in veterinary students.

The first year veterinary students started the academic year as the least sleepy out of all the students. However, even at this point they had what is considered poor sleep quality and higher than normal daytime sleepiness. Over the course of the academic year, their sleep quality and daytime sleepiness scores worsened, coming closely in line with students in other phases of their education. The authors propose that the first year students enter the curriculum as the most rested due to a summer break between undergraduate and veterinary studies with little or no academic responsibilities. The observation of decreasing sleep quality and increasing daytime sleepiness is thought to be a result of the veterinary medical curriculum and lifestyle taking its toll over the course of their first year of instruction.

The second and third year veterinary students were very predictable and had uniformly poor sleep quality and high daytime sleepiness throughout the academic year. The authors found it interesting that there was little variation between the beginning and end of the academic year despite both classes theoretically having not been involved in structured academic pursuits over the summer break. It is possible that these students had simply slipped into uniformly poor sleep habits or that they were more involved in veterinary pursuits than the authors had thought.

Fourth year veterinary students were, unsurprisingly to the authors, the most sleepy and had the poorest sleep quality at the start of the year. This is likely due to them having already spent four to 5 months in time demanding clinical clerkships over the previous summer. What the authors found extremely interesting is that unlike the three other classes, the fourth year students sleep quality and daytime sleepiness improved significantly over the academic year. The authors attribute this to a number of factors including improving ability of dealing with a clinical schedule as the year progresses, passing of stressful milestones such as the national licensing examination, and increasing student morale with the approach of graduation.

When compared to other professional students and students in science fields, veterinary students were similarly sleepy. Human medical, dental, and pharmacy students in several different studies reported similar global PSQI scores with mean scores ranging from 6 to 8 (17, 25, 26). This corresponds closely to the findings for this study.

While this study furthers the understanding of overall veterinary student sleep health, it does have limitations. First and foremost is that the results are directly applicable only to this specific program. Veterinary medical education curricula are individually tailored by each institution and no two are identical although there may be similarities. However, these findings combined with those in the previously referenced study do indicate that this is an issue that should be investigated at other institutions (21). Secondly, PSQI and ESS are very general measures of overall sleep health and do not reveal specific objective data about sleep habits and hygiene. They are limited both as self-reported results which may be inaccurate and influenced by mood or stress. The authors decided not to collect data on student moods or stress levels as the PSQI and ESS combined survey was judged to be long and it was thought that trying to collect additional information would significantly decrease student response rates. Lastly, more detailed demographic information was not collected and so no correlations could be made based on demographics. As veterinary school is a rigid cohort system and has an extremely homogeneous population, the authors felt it unlikely that any statistically significant information would be generated by asking more demographic questions.

While this study sets the idea that poor sleep health can occur in veterinary students, further research is needed to better characterize both individual students and other curricula. Possibilities for further investigation include repeating the study at other colleges of veterinary medicine with different curriculum models to compare differences. A longitudinal cohort study following a single class of students through all 4 years would also give data as to differences between individual classes sleep experiences. A third possibility would be investigating more objective measures of individuals' sleep or sleepiness such as actual time spent sleeping and reaction time testing.

The impact of this study as the first to observe sleep quality and daytime sleepiness of veterinary students over an academic year cannot be understated. The authors have confirmed that veterinary students almost universally have poor sleep quality and above average to excessive daytime sleepiness. Further investigation is necessary to more completely explain some of the trends observed and suggest what resources should be allocated or changes made to veterinary medical education to improve student sleep. As a key component to the discussion of personal wellness in veterinary medical education, poor student sleep health cannot be ignored.

In conclusion, it has been demonstrated that veterinary students have poor sleep quality and above average to excessive daytime sleepiness. Continued investigation and potential intervention is necessary to further characterize and improve veterinary student sleep health.

## Ethics Statement

This study was carried out in accordance with the recommendations of the Virginia Tech Institutional Review Board, Protocol #17-712, with written informed consent from all subjects. All subjects gave written informed consent in accordance with the Declaration of Helsinki. The protocol was approved by the Virginia Tech Institutional Review Board.

## Author Contributions

All authors (MN, LB-W, TS, and SB) have provided the following contributions to the manuscript:

- Substantial contributions to the conception or design of the work; or the acquisition, analysis, or interpretation of data for the work;
- Drafting the work or revising it critically for important intellectual content;
- Final approval of the version to be published;
- Agreement to be accountable for all aspects of the work in ensuring that questions related to the accuracy or integrity of any part of the work are appropriately investigated and resolved.

## Conflict of Interest Statement

The authors declare that the research was conducted in the absence of any commercial or financial relationships that could be construed as a potential conflict of interest.
